# The influence of basic public health service project on maternal health services: an interrupted time series study

**DOI:** 10.1186/s12889-019-7207-1

**Published:** 2019-06-26

**Authors:** Pengyu Zhao, Yifan Diao, Lili You, Shichao Wu, Li Yang, Yuanli Liu

**Affiliations:** School of Public Health, Chinese Academy of Medical Science & Peking Union Medical College, No.5 Dongdansantiao, 100730 Beijing, People’s Republic of China

**Keywords:** Maternal mortality ratio, Maternal health services, Longitudinal study

## Abstract

**Background:**

Reducing maternal mortality remains a global priority. In 2000, the United Nations Member States pledged to work towards a series of Millennium Development Goals (MDGs), in which the fifth target was to reduce maternal mortality ratio by 75% from 1990 to 2015. The Chinese government introduced Basic Public Health Service project in 2009 to the further improvement of maternal health services and reduction in maternal mortality. China had achieved the goal of MDG5 1 year ahead of the schedule in 2014, but the effects of the project on reducing maternal mortality were rarely evaluated with robust methods.

**Methods:**

We conducted a longitudinal study on maternal mortality ratio by extracting mortality data from the National Maternal Mortality Surveillance System (1991–2016) and maternal health services measures from the China health statistic yearbook (2001–2016). We utilized the segmented linear regression model to assess changes and trends of maternal mortality ratio and maternal health services before and after the introduction of Basic Public Health Service project. Pearson correlation analysis was conducted to measure the strength of association between the maternal mortality ratio and maternal health services.

**Results:**

The yearly trend change of national maternal mortality ratio was − 1.76 (*p* < 0.01) after the introduction of Basic Public Health Service project in 2009, while the yearly trend change of maternal health record establish rate, prenatal examination rate, postpartum visit rate was 0.77 (*p* < 0.01), 0.61 (*p* < 0.01) and 0.83 (*p* < 0.01) separately. The negative correlations were also found between national maternal mortality ratio and prenatal examination rate (*r* = − 0.95, *p* < 0.01), maternal health record establish rate (*r* = − 0.93, *p* < 0.01) and postpartum visit rate (*r* = − 0.92, *p* < 0.01).

**Conclusions:**

The Basic Public Health Service project was found to be associated with the improvements in the maternal health services and reduction in maternal mortality. The design and implementation of the project may serve as a positive example for other developing countries. Continued monitoring and assessment of project effects should be stressed.

**Electronic supplementary material:**

The online version of this article (10.1186/s12889-019-7207-1) contains supplementary material, which is available to authorized users.

## Background

It has been estimated that 275,288 maternal deaths occurred worldwide in 2015 [1]. Reducing maternal mortality is considered a global priority since many maternal deaths were preventable. According to the World Health Organization (WHO) report, approximately 800 women would die each day because of preventable causes related to pregnancy and childbirth [2], such as hypertension, infection, severe bleeding. The United Nations (UN) Member States pledged to work towards a series of Millennium Development Goals (MDGs) in 2000 [3], including the target of reducing maternal mortality ratio by 75% from 1990 to 2015 (MDG 5-Target 5.A) [4]. Since more than 99% of the maternal deaths occurred in developing countries [3], the reduction of maternal mortality in developing countries was essential in achieving the MDG 5.

Chinese government endorsed the MDG5 in 2000, and promised to reduce the MMR to 22 per 100,000 livebirths by 2015 [5]. In China, maternal deaths were found to be associated with accessibility of health services, socio-economic backgrounds, cultural factors, biomedical and reproductive factors [6–9]. Chinese government had issued a number of policies targeted at the main causes of maternal deaths, such as “Reducing Maternal Mortality and Eliminating Neonatal Tetanus” in 2000 and the introduction of New Cooperative Medical Scheme (NCMS) in 2003 [10].

From 1990 to 2008, the national MMR in China had dropped 64% (from 95.0 to 34.2 deaths per 100,000 livebirths) [11]; However, the previous policies and interventions may have some limitations. For example, the “Reducing Maternal Mortality and Eliminating Neonatal Tetanus” program was mainly focused on improving hospital delivery rate in a few rural areas in the western and central part of China [12] and the New Cooperative Medical Scheme (NCMS) only focus on limited in-hospital medical insurance for rural residents to reduce their out-of-pocket payments compared to urban residents [13]. In fact, maternal health services should be accessible throughout the pregnancy, rather than only in-hospital time [14]. Early screening and identification of high-risk pregnancy are more likely to rely on community health services. Furthermore, although the national MMR has witnessed huge decline, the gap of MMR between rural and urban areas still exists with respect to the inequality and inadequate of health service deliveries [6, 15]. The MMR in urban and rural in 2008 were 29.2 and 36.1 deaths per 100,000 livebirths, respectively [11]. In such context, with the aims of improving health service continuity and regional equality, the Chinese government launched the Basic Public Health Service (BPHS) project in 2009 [16]. The government-funded project included 9 service categories, one of which was improving maternal health services. It included the establishment of maternal health record, prenatal examinations [17] and postpartum visits [18], which covered almost all key factors throughout pregnancy that could reduce MMR. To address the rural-urban disparities in maternal health services, the BPHS project required equal quality and quantities of maternal health services during the implementation, since maternal health providers were all trained according to national standards and required to deliver same number of maternal services for rural and urban residents. By the year of 2015, the national MMR had decreased to 20.1 per 100,000 livebirths (urban area: 19.8; rural areas: 20.2) [19]. The MDG5 target in reducing China’s MMR was achieved, and the MMR gap between urban and rural areas was narrowed.

Chinese government had made great progress in reducing MMR, while the effects of the implementation of BPHS project were rarely evaluated with robust methods. This paper aims at assessing the effects of BPHS project on reducing MMR in China. Specifically, the annual maternal health record establishment rate, prenatal examination rate, postpartum visit rate before and after the implementation of BPHS project will be measured and their association with MMR will also be explored. Such information would be useful for health administrators to design and implement health projects aiming at reducing MMR and disparities in maternal health services in China and other developing countries.

## Methods

### Study design

We conducted a longitudinal study about national MMR from 1991 to 2016, and maternal health service rate in BPHS project, including maternal health record establishment rate, prenatal examination rate and postpartum visit rate from 2001 to 2016 in China with time-series design of before and after assessments.

### Data collection

The maternal health services in BPHS project included health management in early, middle and late pregnancy periods and the postpartum visit. Firstly, during the first 13 weeks of pregnancy, the first prenatal examination of the mother is carried out by the medical staff at the community health center or township health center where the pregnant woman lives and the health record of mother and children was established at this point. The first prenatal examination cover an inspection of family history, previous history, gynecologic examination, blood routines, urine routines, liver function, kidney function, blood type, hepatitis B examination and so on. The medical staff would also offer guidance and as well as the lifestyle, psychological and nutritional care for the mother in order to prevent the adverse effects of teratogenic factors and disease factors on embryos. For the middle pregnancy health management, the medical staff provide two health education and examination visits for the mother during their pregnancy, once at 16–20 weeks and another at 21–24 weeks. The inspection includes the height of uterine fundus, abdominal girth, position of the fetus, fetal heart rate (times/minute), blood pressure, hemoglobin, urine protein and so on. On the top of that, in late pregnancy period, the medical staff would also provide two health education and examination visits for the mother, one at 28–36 weeks and the other at 37–40 weeks. The examination content is the same as the middle pregnancy health management. Meanwhile, the self-monitoring method is also carried out to promote natural childbirth and breast feeding. Pregnant women who are found to have pregnancy risk factors, contraindications or abnormal examination results at any stage or visit would be referred to a secondary or tertiary health institutions for further diagnoses and treatments. Finally, the postpartum visit was conducted within 28 days postpartum. The visit content includes puerperal health management, breast feeding guidance, neonatal care instructions, health education and so on in order to reduce maternal risks of puerperal infection, postpartum hemorrhage, and postpartum depression.

### Data sources

The national MMR, urban MMR and rural MMR data were extracted from National Maternal Mortality Surveillance System (NMMSS) which set up by the Chinese government in 1989. The sampling unit of the system was at the county (district) level. A total of 336 surveillance spots (126 urban areas and 210 rural areas), covering 31provinces, were selected to record the changes in MMR and the main cause of the maternal death. Livebirths and maternal death data were collected by trained officials and verified by government administrators. In our study, the national MMR data between 1991 and 2016 were extracted and calculated. Maternal health services data in BPHS project were extracted from National Health Statistics Yearbook, including 3 indexes: maternal health record establishment rate, prenatal examination rate and postpartum visit rate. Every province collects and verifies these indexes and uploads the data to the Ministry of Health each year. The data is then presented in the National Health Statistic Yearbook published by the Ministry of Health annually [20]. In our study, the 3 BPHS project maternal health service related indexes were extracted from 2001 to 2016.

### Outcome measures

The official definition of maternal death of WHO is the death of a woman who are pregnant or within 42 days of pregnancy termination, irrespective of the duration and the site of pregnancy, from any cause related to or aggravated by pregnancy or its management, but not from accidental or incidental cause. The causes of maternal death were classified as direct obstetric causes and indirect obstetric causes. In this study, we defined MMR as the maternal death in total and did not distinguish between direct and indirect obstetric deaths. The following indicators were employed to measure the results of maternal health services in BPHS project. The maternal health record establishment rate is defined as the proportion of mothers who have established health records by the professionals during the first 13 weeks of pregnancy among all the mothers resulting in livebirths. The prenatal examination rate is defined as the proportion of mothers who have accepted prenatal examinations at least once during pregnancy among all the mothers resulting in livebirths. The postpartum visit rate is defined as the proportion of mothers who accepted professional postpartum visits at least once among all mothers resulting in livebirths.

### Statistics analyses

We analyzed the time series data using a segmented linear regression model with statistical software (SPSS, Version 21.0) to access changes in levels and trends of national MMR, maternal health record establishment rate, prenatal examination rate and postpartum visit rate before and after the introduction of BPHS project. Interrupted time series analysis can control for auto-correlated errors, and can also adjust for potential serial correlation of the data [21, 22]. We regarded the year of 2009 as the intervention time point for the implementation of BPHS project. Segmented linear regression divided the time series into pre- and post-2009 segments. We then compared the changes in trends and levels of national MMR, maternal health record establishment rate, prenatal examination rate and postpartum visit rate before and after the introduction of BPHS project. Pearson correlation analysis was conducted to measure the correlation between MMR and maternal health record establishment rate, prenatal examination rate and postpartum visit rate. The statistical significance level was set at alpha = 0.05.

## Results

The yearly national MMR, urban MMR and rural MMR have their own variation trend from 1991 to 2016 (Additional file 1). The national MMR ranged from 34.2 to 80.0 per 100,000 livebirths during 1991–2008, which reduced to a range of 19.9–31.9 per 100,000 livebirths during 2009–2016 after the introduction of BPHS project. The MMR in urban and rural areas ranged between 29.20–46.3 per 100,000 livebirths and 36.1 to 100 per 100,000 livebirths respectively during 1991–2008, which went down to a range of 19.5–26.6 per 100,000 livebirths and 20.0–34.0 per 100,000 livebirths during 2009–2016. Maternal health service indicators including maternal health record establish rate, prenatal examination rate and postpartum visit rate also shows different variation trend from 2001 to 2016 (Additional file 2). The maternal health record established rate and prenatal examination rate ranged between87.6–89.4 and 89.7–91.0 respectively before the introduction of BPHS project, and went up to a range of 90.9–96.6 and 92.2–96.6 after the intervention. Postpartum visit rate ranged from the lowest of 85.4 to the highest of 87.2 before the introduction of the BPHS project, and increased to the range of 88.7–94.6 after the intervention.

**Fig. 1:**
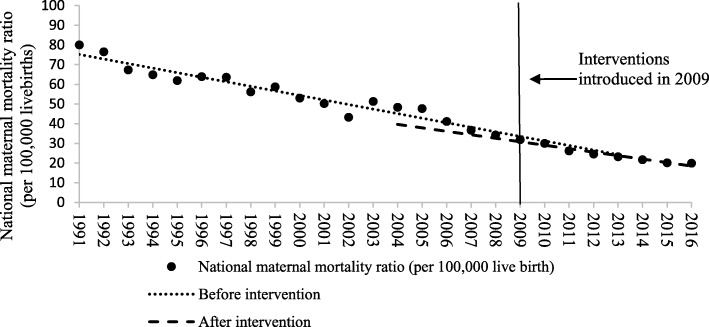
Level and trend changes of national MMR before and after the interventions

**Fig. 2:**
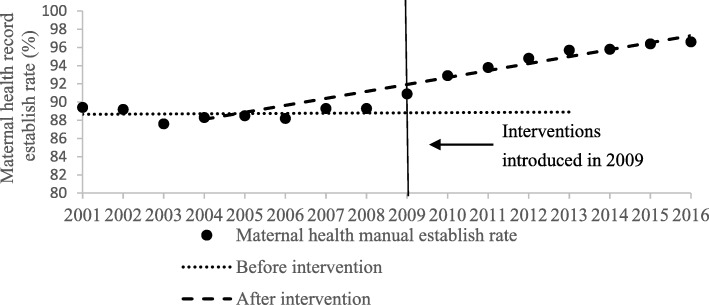
Level and trend changes of maternal health record establish rate before and after the interventions

**Fig. 3:**
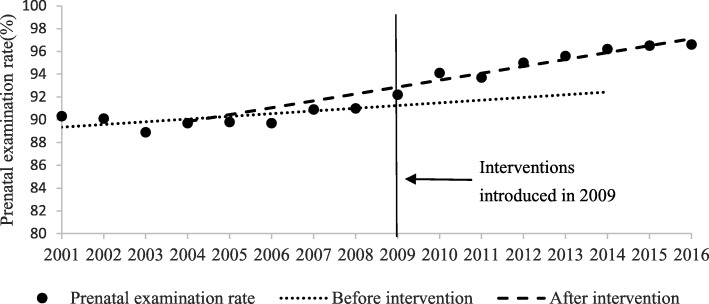
Level and trend changes of prenatal examination rate before and after the interventions

**Fig. 4:**
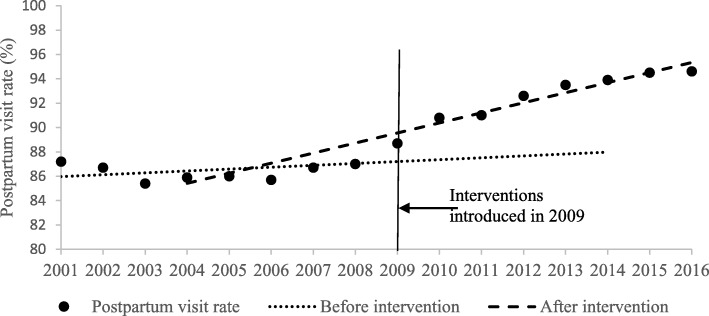
Level and trend changes of postpartum rate before and after the interventions

As presented in Table 1 and Figs. 1, 2, 3 and 4, the segmented regression results show that the trend of the yearly national MMR was − 1.76 (95%CI: − 2.73- -0.79; *p* < 0.01) after the interventions of BPHS project in 2009, which indicate a yearly decline of 1.76 maternal death per 100,000 livebirths. For the maternal health record establish rate, prenatal examination rate and postpartum visit rate, the trend slowly increased before the introduction of BPHS project in 2009. There was an immediate increase in maternal health record establish rate (2.37, 95%CI: 0.86–3.88, *p* = 0.01), prenatal examination rate (1.74, 95%CI: 0.45–3.02, *p* = 0.01) and postpartum visit rate (2.42, 95%CI: 0.85–3.99, *p* = 0.01) respectively when the interventions were introduced (Table 1 and Figs. 1, 2, 3 and 4). The trend of all the rates above show rapid increases of post- intervention, with all results being statistically significant.

**Table 1:** Estimated level and trend changes of national MMR, maternal health record establish rate, prenatal examination rate and postpartum visit rate before and after the interventions

Outcome variables		β	95%CI		*P-*value
National MMR	Intercept	77.45	/	/	/
	Baseline trend	−2.31	−2.6	−2.03	**< 0.01**
	Level change	−3.20	−8.86	2.46	0.254
	Trend change	−1.76	−2.73	−0.79	**< 0.01**
Maternal health record establish rate	Intercept	88.63	/	/	/
	Baseline trend	0.02	−0.21	0.25	0.84
	Level change	2.37	0.86	3.88	**0.01**
	Trend change	0.77	0.54	1.00	**< 0.01**
Prenatal examination rate	Intercept	89.44	/	/	/
	Baseline trend	0.14	−0.06	0.33	0.16
	Level change	1.74	0.45	3.02	**0.01**
	Trend change	0.61	0.41	0.80	**< 0.01**
Postpartum visit rate	Intercept	86.35	/	/	/
	Baseline trend	0.00	−0.24	0.23	0.97
	Level change	2.42	0.85	3.99	**0.01**
	Trend change	0.83	0.57	1.09	**< 0.01**

Bold signifies statistically significant coefficient (*P* < 0.05)

The Pearson correlation analysis results indicated a strong negative correlation between national MMR and maternal health record establish rate, prenatal examination rate and postpartum visit rate. The correlation between MMR and prenatal examination rate was the strongest (*r* = − 0.95, *p* < 0.01), followed by the correlations between MMR and maternal health record establish rate (*r* = − 0.93, *p* < 0.01) and postpartum visit rate (*r* = − 0.92, *p* < 0.01).

## Discussion

In this study, we can see a significant decreasing trend in national MMR and increasing trend in maternal health service volumes after the introduction of BPHS project in 2009. Since the national MMR decreased to 21.7 per 100,000 livebirths and achieved the MDG5 target of reducing maternal mortality to less than 22 per 100,000 livebirths 1 year ahead of schedule, the introduction of BPHS project might have played an important role in this achievement. More importantly, both urban and rural areas achieved the MDG5 target in 2015 with an MMR of 19.8 per 100,000 and 20.2 per 100,000 livebirths respectively which indicates the gap of inequalities and disparities of maternal health services between urban and rural areas were narrowed. The effectiveness of BPHS project confirmed the importance of political will and government commitment in improving maternal health and reducing maternal mortality [23].

Compared with previous policies and interventions, the BPHS project had a national coverage and abundant government funding. The maternal health services in BPHS project covered almost all key factors throughout pregnancy that could reduce maternal mortality [9, 24]. Prenatal examination helped to identify high-risk mothers and sent them to higher specialized hospital for early treatment and to achieve secondary prevention. In addition, the medical staff would keep visiting mothers after the treatment to ensure maternal health. With the introduction of the second-child policy in 2015, the number of mothers, especially late age pregnancy would increase sharply. In such context, the implementation of prenatal examination and prenatal visit was especially challenging and important. Furthermore, the postpartum visits were also important in maternal health, it had been reported by WHO that high-quality postpartum care could have an important role in decreasing mothers’ and infants’ mortality and morbidity rate [25]. Meanwhile, the incidence of postpartum depression rate had also increased in recent years in China and the postpartum visits were supposed to help the prevention and identification of postpartum depression [26]. Both prenatal care and postpartum care played a major role in the reduction of maternal morbidity and mortality [27]. Researches in developing countries such as Nigeria, Sierra Leone, Ethiopia all showed low rates of prenatal care and postpartum visit, and high maternal mortality ratio, which indicated the potential significance of maternal care [28–30] Our results were in line with these findings, showing significantly negative correlations between MMR and prenatal examination rate, maternal health record establish rate, and postpartum visit rate.

The regression analysis showed the effectiveness of BPHS project on improving maternal health services volume throughout the country. There were statistically significant immediate effects on improving prenatal examination rates, maternal health record establish rates and postpartum visit rates. Statistically significant long-term increasing effects on these maternal health services results were also shown, since it kept a slow increasing trend or even no increasing before the introduction of the project. It implies that the maternal health services package of the BPHS project was not a one-time campaign but rather resulted in longitudinal benefit, essential for improving maternal health services.

The regression results of national MMR showed both significant descending trend before and after the introduction of BPHS project, but the immediate effects on reducing national MMR was not statistically significant and the descending trend of national MMR was slower compared with the trend before the introduction of BPHS project. The slower decreasing trend may be due to the boundary effect. It had been reported that the national MMR is more likely to be effected by rural MMR [31, 32], which could be also explained in Chinese condition. MMR in rural areas presented a more rapid decreasing trend from 1991 to 2009 which decreased from 100.0 to 34.0 per 100,000 livebirths and then decreased from 34.0 to 20.0 per 100,000 livebirths from 2009 to 2016. It is difficult to keep the same rapid decline trend when the rate is already at a low level. Compared with urban areas, where it took 22 years to reduce MMR from 38.5 to 19.8 from 1993 to 2005, it took only 8 years for rural areas to reduce their MMR from 34.0 to 20.0 from 2009 to 2016 after the introduction of BPHS project. These findings provide evidence in supporting of the effectiveness of improving maternal health services and MMR reduction.

While China had achieved the MDG5 goal by the end of 2015, many developing countries, especially many African countries still have high MMR levels. The BPHS project in China provides an example for other developing nations on how to carry out continuous, quality maternal health services with the support of relevant evidence. Firstly, the government afforded all the costs of prenatal examinations, prenatal visits and postpartum visits. The community health center, township health center and mothers themselves did not have to bear any cost. This approach not only reduced the financial pressures on primary medical institutions but also made medical staffs more motivated to provide maternal services. Secondly, BPHS project covered all maternal populations, regardless of their age, socioeconomic status, or urban or rural household registrations. This approach increased the equality and demand for services, and resulted in overall better maternal healthcare. Thirdly, the relevant maternal services provided by medical staffs including doctors and pubic physicians in community centers and townships, were preventative in nature, such as health examinations and identifying higher risk patients, so that they could be sent to specialized hospitals for timely and quality treatment. This approach also reflects the effective combination of population strategy and high-risk strategy.

There are several limitations to our present study. First, we could not get detailed information on the cause of maternal death, which limited our ability to analyze the risk factors of the maternal mortality in order to advise future policies for control MMR. Next, this study focused on the trend change and level change of national MMR and maternal health services volume after the introduction of BPHS project, other crucial factors relating to maternal mortality such as health service quality [33, 34] and emergency obstetric care [35] could not be measured and involved in our study. Finally, due to data accessibility, we chose to focus our analysis at the national level. Therefore, some provincial-level differences in how the project was implemented could not be demonstrated in this study. Further data collection could pay attention to the cause of maternal death, information related to other interventions, and differences of provincial implementation of projects if the provincial data are available for more comprehensive and detail analysis and evaluation.

## Conclusion

The evidence generated by the above methods show that China has achieved the MDGs goals in reducing MMR. The BPHS project, a well-designed and carefully arranged nation-wide project by the government, played an important role in improving maternal health services and reducing MMR. This case study sets an effective example for other developing countries to implement organizational and structural changes in order to reduce MMR.

## Additional files

Additional file 1: The yearly national MMR, urban MMR and rural MMR during 1991–2016 (per 100,000 livebirths). (DOCX 14 kb)

Additional file 2: The yearly maternal health record establish rate, prenatal examination rate and postpartum visit rate from 2001 to 2016.(%). (DOCX 12 kb)

## Data Availability

The datasets generated and analyzed during the current study are available in the official website of National Health Commission of the People’s Republic of China (http://www.nhc.gov.cn), and the raw data generated and analyzed are also included as supplementary files.

## Abbreviations

BPHS: Basic Public Health Service
MDG: Millennium Development Goals
MMR: Maternal mortality ratio
NMMSS: National Maternal Mortality Surveillance System
WHO: World Health Organization

## References

[1] Kassebaum NJBA, Coggeshall MS (2016). Global, regional, and national levels and causes of maternal mortality during 1990-2013: a systematic analysis for the global burden of disease study 2013. Lancet.

[2] Alkema L, Chou D, Hogan D, Zhang S, Moller A-B, Gemmill A, Fat DM, Boerma T, Temmerman M, Mathers C (2016). Global, regional, and national levels and trends in maternal mortality between 1990 and 2015, with scenario-based projections to 2030: a systematic analysis by the UN maternal mortality estimation inter-agency group. Lancet.

[3] WHO U, UNFPA, World (2015). Bank Group and the United Nations population division: trends in maternal mortality: 1990 to 2015.

[4] Sachs JD, McArthur JW (2005). The millennium project: a plan for meeting the millennium development goals. Lancet.

[5] Foundation CDR. China human development report 2005. Beijing; 2005.

[6] Du Q, Nass O, Bergsjo P, Kumar BN (2009). Determinants for high maternal mortality in multiethnic populations in Western China. Health Care Women Int.

[7] Harris AZY, Liao H (2010). Challenges to maternal health care utilization among ethnic minority women in a resource-poor region of Sichuan Province, China. Health Policy Plan.

[8] You F, Huo K, Wang R, Xu D, Deng J, Wei Y, Shi F, Liu H, Cheng G, Zhang Z (2012). Maternal mortality in Henan Province, China: changes between 1996 and 2009. PLoS One.

[9] Liang J, Dai L, Zhu J, Li X, Zeng W, Wang H, Li Q, Li M, Zhou R, Wang Y (2011). Preventable maternal mortality: geographic/rural-urban differences and associated factors from the population-based maternal mortality surveillance system in China. BMC Public Health.

[10] Wang Y (2007). Development of the new rural cooperative medical system in China. Chin World Econ.

[11] Jiang FHL, Rakofsky J, Liu T, Wu S, Zhao P, Hu G, Wan X, Liu H, Y1 L, Tang YL. Sociodemographic characteristics and job satisfaction of psychiatrists in China: results from the first Nationwide survey. Beijing Psychiatr Serv. 2018. 10.1176/appi.ps.201800197 30301449

[12] Liang J, Li X, Kang C, Wang Y, Kulikoff XR, Coates MM, Ng M, Luo S, Mu Y, Wang X (2019). Maternal mortality ratios in 2852 Chinese counties, 1996-2015, and achievement of millennium development goal 5 in China: a subnational analysis of the global burden of disease study 2016. Lancet.

[13] Long Q, Zhang T, Xu L, Tang S, Hemminki E (2010). Utilisation of maternal health care in western rural China under a new rural health insurance system (new co-operative medical system). Trop Med Int Health.

[14] Ekman B, Pathmanathan I, Liljestrand J (2008). Integrating health interventions for women, newborn babies, and children: a framework for action. Lancet.

[15] Gao Y, Kildea S, Barclay L, Hao M, Zeng W (2009). Maternal mortality surveillance in an inland Chinese province. Int J Gynaecol Obstet.

[16] Zhao YHZ, Jian WU (2013). Impact on the performance of health workers adopted performance-related contracts in the provision of basic public health service at village and township levels. Iran J Public Health.

[17] Berhan Y, Berhan A (2014). Antenatal care as a means of increasing birth in the health facility and reducing maternal mortality: a systematic review. Ethiop J Health Sci.

[18] Spelke B, Werner E (2018). The fourth trimester of pregnancy: committing to maternal health and well-being postpartum. R I Med J (2013).

[19] China MoHoTPsRo (2011). China health statistics yearbook. [in Chinese].

[20] Fang J, Kaufman J (2008). Reproductive health in China: improve the means to the end. Lancet.

[21] Wagner AK, Soumerai SB, Zhang F (2002). Segmented regression analysis of interrupted time series studies in medication use research. J Clin Pharm Ther.

[22] Lagarde M (2012). How to do (or not to do) ... Assessing the impact of a policy change with routine longitudinal data. Health Policy Plan.

[23] Shiffman J (2007). Generating political priority for maternal mortality reduction in 5 developing countries. Am J Public Health.

[24] Averbach S, Kakaire O, Kayiga H, Lester F, Sokoloff A, Byamugisha J, Dehlendorf C, Steinauer J (2017). Immediate versus delayed postpartum use of levonorgestrel contraceptive implants: a randomized controlled trial in Uganda. Am J Obstet Gynecol.

[25] WHO (1999). Postpartum Care of the Mother and Newborn: a practical guide. Birth.

[26] Gjerdingen DK, Yawn BP (2007). Postpartum depression screening: importance, methods, barriers, and recommendations for practice. J Am Board Fam Med.

[27] Dhakal PSM, Baral D (2018). Factors affecting the place of delivery among mothers residing in Jhorahat VDC, Morang, Nepal. Int J Community Based Nurs Midwifery.

[28] Fawole AO, Shah A, Fabanwo AO (2012). Predictors of maternal mortality in institutional deliveries in Nigeria. Afr Health Sci.

[29] Koroma MM, Kamara SS, Bangura EA, Kamara MA, Lokossou V, Keita N (2017). The quality of free antenatal and delivery services in northern Sierra Leone. Health Res Policy Syst.

[30] Muchie KF (2017). Quality of antenatal care services and completion of four or more antenatal care visits in Ethiopia: a finding based on a demographic and health survey. BMC Pregnancy Childbirth.

[31] Ronsmans C, Graham WJ (2006). Maternal mortality: who, when, where, and why. Lancet.

[32] Bartlett LA, Mawji S, Whitehead S, Crouse C, Dalil S, Ionete D, Salama P (2005). Where giving birth is a forecast of death: maternal mortality in four districts of Afghanistan, 1999–2002. Lancet.

[33] G L: Beyond the numbers: reviewing maternal deaths and complications to make pregnancy safer, vol. 67; 2003. 10.1093/bmb/ldg009 14711752

[34] Ivers N, Jamtvedt G, Flottorp S, Young JM, Odgaard-Jensen J, French SD, O’Brien MA, Johansen M, Grimshaw J, Oxman AD. Audit and feedback: effects on professional practice and healthcare outcomes. Cochrane Database Syst Rev. 2012;(6):CD000259. 10.1002/14651858.CD000259.pub3 PMC11338587 22696318

[35] Paxton A, Maine D, Freedman L, Fry D, Lobis S (2005). The evidence for emergency obstetric care. Int J Gynaecol Obstet.

