# Supplementary material for: The influence of basic public health service project on maternal health services: an interrupted time series study
Source: BMC Public Health. 2019 Jun 26;19:824. doi: 10.1186/s12889-019-7207-1 (PMC6595598; doi:10.1186/s12889-019-7207-1)
Supplement: Supplementary file 1 — The yearly national MMR, urban MMR and rural MMR during 1991–2016 (per 100,000 livebirths). (DOCX 14 kb) [file 12889_2019_7207_MOESM1_ESM.docx]

**Additional file 1**

The yearly national MMR, urban MMR and rural MMR during 1991-2016 (per 100,000 livebirths)

| Year | National MMR | Urban MMR | Rural MMR |
| --- | --- | --- | --- |
| 1991 | 80.0 | 46.3 | 100.0 |
| 1992 | 76.5 | 42.7 | 97.9 |
| 1993 | 67.3 | 38.5 | 85.1 |
| 1994 | 64.8 | 44.1 | 77.5 |
| 1995 | 61.9 | 39.2 | 76.0 |
| 1996 | 63.9 | 29.2 | 86.4 |
| 1997 | 63.6 | 38.3 | 80.4 |
| 1998 | 56.2 | 28.6 | 74.1 |
| 1999 | 58.7 | 26.2 | 79.7 |
| 2000 | 53.0 | 29.3 | 69.6 |
| 2001 | 50.2 | 33.1 | 61.9 |
| 2002 | 43.2 | 22.3 | 58.2 |
| 2003 | 51.3 | 27.6 | 65.4 |
| 2004 | 48.3 | 26.1 | 63.0 |
| 2005 | 47.7 | 25.0 | 53.8 |
| 2006 | 41.1 | 24.8 | 45.5 |
| 2007 | 36.6 | 25.2 | 41.3 |
| 2008 | 34.2 | 29.2 | 36.1 |
| 2009 | 31.9 | 26.6 | 34.0 |
| 2010 | 30.0 | 29.7 | 30.1 |
| 2011 | 26.1 | 25.2 | 26.5 |
| 2012 | 24.5 | 22.2 | 25.6 |
| 2013 | 23.2 | 22.4 | 23.6 |
| 2014 | 21.7 | 20.5 | 22.2 |
| 2015 | 20.1 | 19.8 | 20.2 |
| 2016 | 19.9 | 19.5 | 20.0 |
