# Supplementary material for: The influence of basic public health service project on maternal health services: an interrupted time series study
Source: BMC Public Health. 2019 Jun 26;19:824. doi: 10.1186/s12889-019-7207-1 (PMC6595598; doi:10.1186/s12889-019-7207-1)
Supplement: Supplementary file 2 — The yearly maternal health record establish rate, prenatal examination rate and postpartum visit rate from 2001 to 2016.(%). (DOCX 12 kb) [file 12889_2019_7207_MOESM2_ESM.docx]

**Additional file 2**

The yearly maternal health record establish rate, prenatal examination rate and postpartum visit rate from 2001 to 2016.（%）

| Year | Maternal heath record establish rate | Prenatal examination rate | Postpartum visit rate |
| --- | --- | --- | --- |
| 2001 | 89.4 | 90.3 | 87.2 |
| 2002 | 89.2 | 90.1 | 86.7 |
| 2003 | 87.6 | 88.9 | 85.4 |
| 2004 | 88.3 | 89.7 | 85.9 |
| 2005 | 88.5 | 89.8 | 86.0 |
| 2006 | 88.2 | 89.7 | 85.7 |
| 2007 | 89.3 | 90.9 | 86.7 |
| 2008 | 89.3 | 91.0 | 87.0 |
| 2009 | 90.9 | 92.2 | 88.7 |
| 2010 | 92.9 | 94.1 | 90.8 |
| 2011 | 93.8 | 93.7 | 91.0 |
| 2012 | 94.8 | 95.0 | 92.6 |
| 2013 | 95.7 | 95.6 | 93.5 |
| 2014 | 95.8 | 96.2 | 93.9 |
| 2015 | 96.4 | 96.5 | 94.5 |
| 2016 | 96.6 | 96.6 | 94.6 |
